# Anterior chest wall reconstruction with titanium plate sandwiched between two polypropylene sheets

**DOI:** 10.1007/s11748-012-0048-9

**Published:** 2012-05-19

**Authors:** Keitaro Matsumoto, Isao Sano, Akihiro Nakamura, Shigeyuki Morino, Naoya Yamasaki, Tomoshi Tsuchiya, Takuro Miyazaki, Takeshi Nagayasu

**Affiliations:** 1 Division of Surgical Oncology, Department of Translational Medical Sciences, Nagasaki University Graduate School of Biomedical Sciences, 1-7-1 Sakamoto, Nagasaki, 852-8501 Japan; 2 Department of Surgery, Japanese Red Cross Nagasaki Genbaku Hospital, Nagasaki, Japan; 3 Department of Surgery, Sasebo City General Hospital, Sasebo, Nagasaki Japan

**Keywords:** Chest wall reconstruction, Sarcoma, Sternum, Surgical instruments

## Abstract

Extensive sternal resection carries the risk of difficult reconstruction and surgical complications. A 79-year-old woman underwent sternal resection and reconstruction for sternal chondrosarcoma. However, 18 months after the first operation, she developed six metastatic tumors on the anterior chest wall. She underwent subtotal sternectomy and rib resection, leaving a defect measuring 17 × 14 cm. Reconstruction of the anterior chest wall using a titanium plate sandwiched between two polypropylene mesh sheets is described. This method is potentially applicable to extensive anterior chest resection, and its advantages compared with conventional prostheses are rigidity, flexibility, and usability.

## Introduction

Resection of malignant sternal tumors requires extensive resection of the anterior chest wall. Although various prostheses have been used for anterior chest wall reconstruction, selection of the procedure depends on the surgeons’ experience. The case of a patient who underwent reconstruction of the anterior chest wall using a titanium plate sandwiched between two polypropylene mesh sheets is reported.

## Case report

**Fig. 1:**
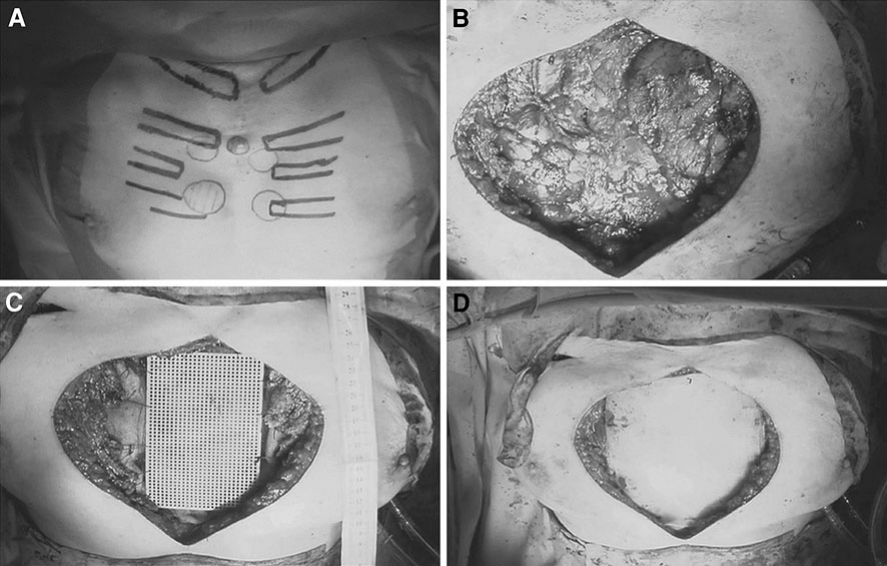
Surgical images. **a** Local recurrent tumors on the chest wall. The skin and subcutaneous segments were marked before surgery. **b** Chest wall defect after subtotal sternectomy and resection of the 1st–5th ribs and costal arch. **c** The middle layer consists of a titanium plate fixed to the manubrium and costal arch, pulled to each rib stump. The lowermost layer is a polypropylene mesh sheet. **d** The uppermost layer consists of a polypropylene mesh sheet fixed to the manubrium and each rib

**Fig. 2:**
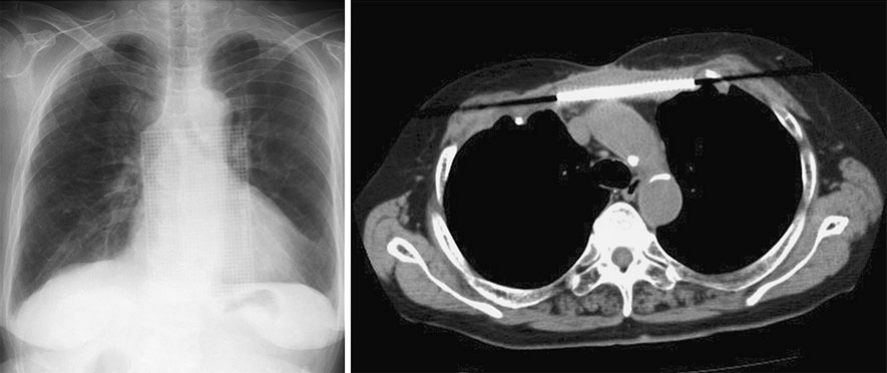
Postoperative chest X-ray and computed tomography scans showing the titanium plates secured to the manubrium and ribs

A 79-year-old woman was referred to our department with a diagnosis of recurrent chondrosarcoma. The first operation for sternal chondrosarcoma included sternal resection and reconstruction with polypropylene mesh and a musculocutaneous flap. However, 18 months after the first operation, computed tomography revealed five tumors located on the anterior chest wall and another tumor located in the subcutaneous tissue on the right chest wall. The tumors were considered metastatic lesions, with no evidence of enlarged mediastinal lymph nodes or distant metastases on radiographic examination. Thus, it was judged that complete resection was possible, and the patient underwent subtotal sternectomy, total resection of the body and partial resection of the manubrium sternii, together with partial resection of the 1st–5th ribs and costal arch, with a surgical margin of more than 1.0 cm for each tumor. This resection left a defect measuring 17 × 14 cm on the anterior chest wall. Reconstruction of the defect was undertaken with a titanium plate (Titanium Mini Mesh Sheet, 01-13155, 132 × 82 mm; thickness 0.5 mm, Stryker Leibinger & Co., Germany) sandwiched between two polypropylene mesh sheets. The lowermost and the uppermost layer consisted of a polypropylene mesh, and the sheet was fixed to the manubrium and each rib with absorbent suture. The middle layer was a titanium plate, which was fixed to the manubrium and costal arch directly by absorbable #2 polyfilament braided suture and pulled toward each rib stump (Fig. 1). The soft tissue covering was sutured directly. No paradoxical movement of the rib cage was noted during respiration in the postoperative period. Histopathological examination of the resected tissue showed recurrent sternal chondrosarcoma in all six tumors. Twelve months after operation, the patient had maintained excellent range of motion without instability or lordosis (Fig. 2).

## Discussion

Sternal tumors are uncommon; however, they are of different pathological types, such as sarcoma and metastatic tumors of the breast, thyroid, kidney, and colon. Such tumors require wide and full-thickness resection for complete removal. King et al. [1] recommended a 4-cm free margin for highly aggressive primary tumors and 2-cm margins for metastatic, benign, or low-grade malignancies to avoid local recurrences. In any case, complete resection of the sternal tumor results in a wide defect on the anterior chest wall.

Various procedures have been used to reconstruct wide defects of the anterior chest wall. The ideal prosthetic material should be easily available, durable, easily usable, adaptable, rigid, resistant to infection, translucent to radiographs, and of low cost. Generally, polypropylene mesh sheets or polytetrafluoroethylene patches (e-PTFE) covered with a musculocutaneous flap are used [2]. However, their rigidity is insufficient to protect intrathoracic organs. Various prostheses have been used, with sufficient rigidity, such as sandwiched polypropylene mesh and stainless steel mesh [3], methyl methacrylate sandwiched between polypropylene mesh [4], titanium plate-supported methyl methacrylate sandwich [5], titanium plate with Gore-Tex^®^ dual mesh [6], and Composix Mesh^™^ [7]. However, methyl methacrylate is not easy to handle and is difficult to adapt to the shape of the patient’s chest. Titanium Mini Mesh Sheet has strong rigidity, no plasticity, translucency to radiography, magnetic resonance imaging (MRI) compatibility, and biocompatibility. We think that the combination of a metal material and a mesh is an appropriate prosthesis, because of its durability, ease of use, adaptability, rigidity, and translucency to radiography. The advantages of the present procedure are based on the easy use of the titanium plate, irrespective of the shape of the defect and the physiological nature of the material. The titanium plate is used to provide protection for intrathoracic organs, while the polypropylene mesh is flexible in both vertical directions and thus allows movement of the chest wall during breathing.

In conclusion, the procedure with the titanium plate sandwiched between two polypropylene meshes achieved good fixation and flexibility. In patients who require extensive anterior chest wall and sternal resection, this technique may be suitable for reconstruction.
